# 42 Midlines for Outpatient Parenteral Antimicrobial Therapy: A Not-So-Safe Alternative to Peripherally Inserted Central Catheters?

**DOI:** 10.1017/ash.2026.10711

**Published:** 2026-06-23

**Authors:** Lisa Parlich, Michelle Campoli, Heather Voss, Christina Silkaitis, Mary Anne Sotelo

**Affiliations:** 1 Northwestern Medicine; 2 Northwestern Medicine McHenry; 3 Northwestern Memorial Healthcare

## Abstract

**Background:** A potential cluster of post-craniotomy surgical site infections was noted by the neurosurgery service in December 2024. A case review revealed a total of five organ space surgical site infections (SSI) identified between October 4th-December 4th, three of which had the same organism isolated, Cutibacterium acnes (C. acnes). **Methods:** Infection Prevention met with Surgical Services, Sterile Processing, Neurosurgery, and Infectious Disease to strategize next steps. After a retrospective review of the five initial SSIs, a case definition was established including patients who underwent a craniotomy procedure and had cultures positive for C. acnes collected upon readmission with signs of infection. An epidemiological curve and line list of cases meeting definition was created. Excluded cases included: C. acnes isolated from the index procedure and unrelated to an infection, isolation of a different organism post craniotomy, and infection post burr hole procedure. Observations of the neurology OR and case setup, pre/intra/post-op workflows and sterile processing practices were completed and staff interviewed. Surgical positioning equipment and instrument instructions for use (IFU) were reviewed. Equipment and implants used in each case were noted and compared to cases without infections to identify similarities. A common DuraGen implant was sent to the Microbiology Laboratory for culturing. The C. acnes isolates were sent to the Molecular Laboratory for genomic typing. **Result:** Altogether, four patients met the case definition. No deficiencies were identified during the observation of the neurology OR and case setup. Staff interviews revealed patients would arrive from the inpatient units prior to procedure with dirty hair and bed linens. Investigation into intra-op practices revealed that key positioning equipment was not being cleaned or disinfected per manufacturer’s IFU. Additionally, a retrospective chart review of index procedures revealed a common DuraGen 2x2 implant was utilized in each case. Subsequent culturing of the DuraGen implant resulted in growth of a Cutibacterium species. Molecular typing of the patient and DuraGen samples revealed two of the four patient samples to be genetically similar, however none of the patients to be genetically similar to the DuraGen sample. **Conclusion:** Quick attention to gaps in the OR and engagement of the leadership team led to improved practices and adherence to IFUs. Since the fourth and final case, there have been no additional C. acnes SSI cases. As a result of the investigation, craniotomy cases have been added to Infection Prevention’s daily surveillance with completion of root cause analyses for any infection,regardless

